# A Review of Artificial Diets for Aphids (Hemiptera: Aphididae)

**DOI:** 10.3390/insects17030326

**Published:** 2026-03-17

**Authors:** Rongrong Gao, Qingqiu Zeng, Ming Zhu, Zhentao Ren, Kun Xue

**Affiliations:** 1 Key Laboratory of Ecological Environment in Ethnic Areas, The State Ethnic Affairs Commission, Minzu University of China, Beijing 100081, China; 2 College of Life and Environmental Sciences, Minzu University of China, Beijing 100081, China; 3 Open Education College, Yunnan Open University, Kunming 650000, China; 4 Institute of Food and Nutrition Development, Ministry of Agriculture and Rural Affairs, Beijing 100081, China

**Keywords:** aphids, artificial diets, insect nutrition, digestive enzymes, pest control

## Abstract

Aphids are among the most significant agricultural pests worldwide. Artificial diets are a critical foundation for aphid physiological and biochemical research and the development of pest control technology. However, their phloem sap-feeding habits, extraoral digestion characteristics, and host specificities pose numerous challenges to the development of artificial diets for aphids, including population degradation, reduced fecundity during long-term rearing, and a lack of methodological diversity in dietary formulation research. In this review, we summarize the research on artificial diets for aphids, encompassing the history of artificial rearing, rearing methods, and nutritional composition analysis of these diets. Furthermore, we discuss the optimization of diet formulations based on aphid digestive enzymes and symbiotic bacteria. We aim to synthesize successful developments of artificial diets for aphids and extend their application to diverse aphid species. Future development of artificial aphid diets should focus on matching the types and contents of nutritional elements with the digestive enzymes and gut microbes of aphids. It is necessary to develop specific artificial diets for targeted aphid populations rather than merely adopting successful formulations and experience with *Myzus persicae* or *Acyrthosiphon pisum*. In addition, automated and large-scale aphid rearing devices should be developed, and further research on population degradation during long-term aphid rearing is required to explore multifaceted solutions involving nutritional and environmental aspects.

## Simple Summary

Artificial diets have facilitated in-depth physiological research and pest control in aphids. In this review, we summarize the successful artificial diets for aphids, including *Myzus persicae*, *Acyrthosiphon pisum*, *Aphis fabae*, *Macrosiphum euphorbiae*, and *Aphis glycines*. However, many aphid species still cannot survive or reproduce properly on artificial diets. Some key successful approaches for developing artificial diets for aphids are discussed, including (1) optimizing nutrient ratios, (2) precisely adding specific functional nutrients, (3) introducing feeding stimulants and other active growth factors, (4) replacing complex natural components with well-defined substitutes, and (5) exploring future improvements based on aphid digestive enzymes and symbiotic bacteria.

## 1. Introduction

Aphids are among the world’s most economically important agricultural pests, with more than 5100 species widely distributed across temperate regions [1,2]. Holman published a catalog presenting data for 3654 aphid species identified on 11,793 plants from 246 plant families in the Palaearctic region. The study of aphid host plants provides useful information about the ecology, evolution, and distribution of aphid species [3]. The host range of aphids encompasses nearly all families of angiosperms and gymnosperms as well as bryophytes and ferns [4]. Of the 45 major insect pests infesting six primary temperate climate crops (maize, wheat, potatoes, sugar beet, barley, and tomatoes), 26% were aphids [5]. Nearly all primary agricultural crops act as hosts to at least one aphid species, several of which are classified as the world’s most destructive agricultural pests [6]. For instance, *Schizaphis graminum*, *Sitobion avenae*, *Metopolophium dirhodum*, and *Rhopalosiphum padi* are the dominant insect pests infesting wheat-growing regions in China. Wheat aphids are major pests in wheat production in China and worldwide. This reduces wheat yield by over 10% annually, and, in severe infestation years, the reduction can exceed 30% [7]. Aphid infestations resulted in average direct yield losses of 8–16% for peas, 10–13% for wheat, and 5% for potatoes [8].

Artificial diet systems have been widely employed in numerous physiological, biochemical, and toxicological studies on aphid management. These systems facilitate the investigations of insecticidal compounds, insect resistance targets, gene silencing, the gut microbiota, and viral transmission mechanisms. The results of this study provide a fundamental basis for the development of pesticides, transgenic crops, and gene-editing-based products. Aphids feed exclusively on plant phloem sap, a highly specialized nutritional source, and exhibit unique biological traits, including extraoral digestion and honeydew excretion. These characteristics are key factors that have hampered the development of effective artificial diets for aphids. Research on artificial aphid diets dates back to the early 1930s, when Hamilton conducted studies on aphid viral transmission mechanisms [9] and first attempted to rear aphids using membrane-based systems (fish skin or sausage casing) containing artificial diet formulations. In 1956, Parafilm—a 50:50 wax-polyolefin blend—was first used for artificial aphid diet feeding assays; this method enabled the successful detection of virus-carrying aphids and the characterization of viral loss when aphid stylets pierced or traversed inert substrates [10]. Subsequently, landmark breakthroughs were made in the rearing of green peach aphids (*Myzus persicae*) and pea aphids (*Acyrthosiphon pisum*), with these species surviving for several weeks on an artificial diet [11,12]. By the mid-1960s, Mittler and Dadd developed artificial diets that supported the continuous rearing of *M. persicae* for more than 20 generations [13]. During this period, a series of investigations focused on the nutritional requirements, dietary components, rearing apparatuses, and experimental protocols of *M. persicae* and *A. pisum*, which gradually led to the development of optimized artificial diet systems for these two species and provided a theoretical framework for the artificial rearing of other aphid taxa [14,15,16,17,18,19,20,21]. Building on this foundational research, rearing performance was further improved for additional aphid species, including the black bean aphid (*Aphis fabae*), potato aphid (*Macrosiphum euphorbiae*), *Tuberaphis styraci*, soybean aphid (*Aphis glycines*), cotton aphid (*Aphis gossypii*), and grain aphid (*S. avenae*), several of which are now continuously reared for multiple generations on artificial diets [22,23,24,25,26].

However, artificial diets present several challenges, including inferior development and reproductive performance relative to natural host plants, population deterioration during prolonged rearing, and interspecific differences in nutritional requirements among aphid species [27,28,29]. This review summarizes the key aspects, including comparisons of artificial diet formulations for aphids and analyses of dietary components; reconsiders strategies for the development of artificial aphid diets; and provides a reference framework for future research on artificial aphid diets.

## 2. Historical Development of Aphids Reared on Artificial Diets

Considerable variations in rearing performance have been observed during the development of artificial aphid diets. Therefore, research efforts have predominantly focused on the continuous optimization of dietary components.

Since 1962, through the establishment of a basic formula and subsequent multiple modifications—including adjusting amino acid ratios, adding trace metal elements (iron, zinc, manganese, copper) or nucleic acids, and incorporating yeast extract (YE) and sodium acetate—the rearing performance of *M*. *persicae* has gradually improved. Starting with an initial survival rate of only 50% over a two-week period, continuous rearing of more than 20 generations was gradually achieved. With further optimization of diet formulations, aphid populations have been successfully maintained for more than three decades. During diet development, the body weight and reproductive capacity of the aphids gradually improved.

Similarly, *A. pisum* was successfully reared in 1963. Subsequently, by simulating its internal amino acid composition, optimizing metal element concentrations (e.g., Cu^2+^, Fe^3+^, Mn^2+^, Zn^2+^), and supplementing key components such as aromatic amino acids and β-alanyltyrosine, the number of rearing generations gradually increased from an initial two generations to more than 46 generations, with significant improvements in adult body weight and reproductive rate.

Other aphid species have also achieved varying degrees of artificial rearing by adjusting their dietary ingredients based on the formulas of *M. persicae* and *A. pisum*. For example, *A. fabae* can be continuously reared for 21–30 generations. *A. gossypii* exhibits significant host specialization, with some strains being capable of being reared for only 2–3 generations, whereas a specific cotton-adapted strain can reach 23 generations. For *Rhopalosiphum maidis*, *M*. *euphorbiae*, *S*. *avenae*, *T*. *styraci*, *A*. *glycines*, and *Brevicoryne brassicae*, continuous rearing for 3–17 generations have been achieved, with some results approaching the rearing levels observed in natural host plants.

**Figure 1:**
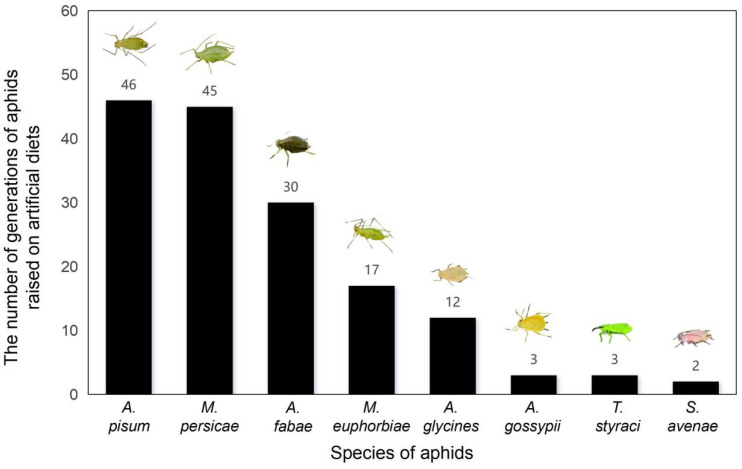
The number of generations of aphids raised on artificial diets.

Several aphid species can reproduce for multiple generations using artificial diets, including *A. pisum* [24], *M. persicae* [30], *A*. *fabae* [21], *M*. *euphorbiae* [31], *A*. *glycines* [32], *A*. *gossypii* [18], *T*. *styraci* [33,34], and *S*. *avenae* [35] (Figure 1).

Research on artificial diets for aphids has significantly improved the number of surviving generations, body weight, and reproductive capacity of various aphid species by continuously optimizing nutritional components and key additives. These studies provide a reliable foundation for the continuous laboratory rearing of aphids. The detailed formulation development process is as follows.

The earliest successful artificial diet for rearing *M*. *persicae* was developed by Mittler and Dadd in 1962, with more than 50% of the aphids surviving for two weeks and producing offspring [11,14]. Subsequently, in 1965, they further optimized the artificial diet formulation; by addressing diet precipitation and improving the amino acid ratio, they maintained aphid survival while increasing both the number of offspring and the adult body weight [16]. A significant breakthrough occurred in 1966; the addition of metal elements such as iron, zinc, manganese, and copper enabled the continuous rearing of *M. persicae* for 20 generations, with further increases in body weight [13]. Some clones of *M. persicae* showed good adaptation to the artificial diet after three generations [36]. In 1967, research demonstrated that nucleic acids contained trace metal micronutrients (iron, zinc, manganese, and copper) and that the addition of nucleic acids yielded growth improvements similar to those observed with direct metal supplementation [20]. Incorporating YE into the artificial diet allowed the rearing of *M. persicae* for 45 generations and increased body weight [30]. In 2020, by adding sodium acetate to the artificial diet and making specific improvements to previous formulations, researchers successfully maintained the continuous rearing of one *M. persicae* genotype for more than 30 years, although this genotype abruptly went extinct in 2008 [29].

Similarly, *A. pisum* was first successfully reared by Auclair and Cartier in 1963. Based on the average concentrations of amino acids and amide compounds in *A. pisum* bodies and honeydew and drawing on the vitamin and salt mixture ratios from *M. persicae* diets, they developed an artificial diet that successfully reared *A. pisum* for two generations, with survival periods of 10–20 days [12]. In 1965, a series of diet optimizations enabled *A. pisum* to survive for three generations (a total of 64 days) and increase its adult body weight [17]. In 1971, a modified formulation based on the 1966 *M. persicae* diet [13] allowed the continuous rearing of *A. pisum* for seven generations [23]. Optimization of the 1963 diet formulation [12,37], combined with further modifications to the inorganic salt composition of its host plant (broad bean), led to the successful rearing of *A. pisum* for 18 generations through the addition of Cu^2+^, Fe^3+^, Mn^2+^, and Zn^2+^ in the form of chloride salts [25]. In 1972, Akey and Beck adjusted the contents of various trace metals, inorganic sulfur, and cholesterol benzoate in the diet, achieving continuous rearing of *A. pisum* for more than 46 generations while improving the reproductive rate and body weight [24]. In 1988, research revealed that supplementing aromatic amino acids and β-alanyltyrosine could overcome tyrosine deficiency caused by its poor solubility [38]. In 1991, researchers formulated two diets by simulating the amino acid compositions of plant sap and *A. pisum* tissues, which increased the reproductive output and body weight of *A. pisum* over a two-generation experimental period [39].

Furthermore, progress has been made in the artificial rearing of other aphid species. In 1967, by incorporating trace metals (iron, zinc, manganese, and copper), *A. fabae* infesting *Rumex* plants were continuously reared for 30 generations, whereas those infesting *Tropaeolum* plants were continuously reared for 21 generations [21]. *A*. *gossypii* was initially only reared for two generations on an artificial diet [18]. However, different host-specialized biotypes of *A. gossypii* exhibit varying adaptability to artificial diets, and the cotton-associated biotype can survive continuously for 23 generations on a specifically formulated diet, whereas most other biotypes tested on the same diet can only be maintained for 2–3 generations [40]. Modifications to the 1963 artificial diet developed for *A*. *pisum* [12], when used to feed *R*. *maidis*, enabled continuous survival for three weeks and successful reproduction [41]. The addition of chlorogenic acid to *A. pisum* diets [23] improved the growth of *M*. *euphorbiae*, allowing its continuous survival for 17 generations [31]. In 2000, based on *A. pisum* diet formulations in 1995–1997 and comparisons of diet pH and the nutritional requirements of *S*. *avenae*, researchers developed a new formulation capable of continuously rearing *S. avenae* for two generations [35]. In 2002, a modified diet formulation based on the amino acid profile of hydrolyzed *A. pisum* tissues [38] allowed for the continuous rearing of *T*. *styraci* for three generations [33]. In 2008, based on a diet of *M. persicae* [42] with adjusted amino acid content, *A*. *glycines* was successfully reared for 12 generations [32]. In 2009, researchers found that using the 1988 *A. pisum* artificial diet [38] to rear *B*. *brassicae* could maintain all life stages of this aphid, with no significant differences compared with rearing on cabbage leaf discs [43]. In 2024, drawing on various existing artificial aphid diet formulations, researchers designed and screened a diet suitable for *A. gossypii* rearing that was simple to prepare and significantly improved the survival rate of *A. gossypii* [44].

One diet often outperforms the other because the contents of several major nutrients reach an optimal level suitable for specific aphid species, such as sucrose and amino acids. In addition, maintaining a wide variety and precise quantification of nutrients in the diet is the key to achieving a good rearing effect on aphids. Therefore, increasing the content of certain major nutrients, such as sucrose or amino acids, can improve the aphid-rearing effect to a certain extent. Owing to the interspecific differences among aphids, the rearing effect of artificial diets is closely related to the aphid species. Diet formulations for most aphid species are based on those originally designed for *M. persicae* or *A. pisum*, and aphid diets modified on this basis naturally yield poor rearing results when used for other aphid species.

Table 1 lists some diet formulations that support rearing aphids for multiple generations. The differences in diet formulas mainly lie in the content of certain nutrients, as detailed in the table below.

## 3. Effects of Different Nutritional Components on Aphid Growth and Development

### 3.1. Carbohydrates

Carbohydrates ingested by aphids primarily include sucrose, glucose, fructose, and maltose. Studies demonstrated that sugars acted as nutritional substrates, feeding stimulants, and osmotic regulators for aphid nutritive solutions [45,46,47,48].

#### 3.1.1. Sucrose

Among these sugars, sucrose has the most pronounced effects on stimulating feeding behavior and regulating osmotic pressure, whereas fructose and glucose play secondary roles in stimulating ingestion. When sucrose was replaced completely or partially with glucose, fructose, or any of the following sugars—cellobiose, galactose, lactose, melezitose, raffinose, ribose, sorbose, or trehalose—in artificial diets, the survival rate of *A. gossypii* decreased sharply. This reduction in survival may result from low palatability or inadequate nutritional value of the substitute sugars [18]. Compared with other sugars, high concentrations of sucrose not only stimulate feeding but also enhance aphid growth and development [25]. Accordingly, sucrose is the most widely used sugar in the formulation of aphid artificial diets.

Sucrose concentration preferences vary not only among aphid species but also among different developmental stages within the same species. For *M. persicae*, an artificial diet containing 10–20% sucrose supports optimal growth of both nymphs and adults, whereas concentrations below 10% lead to significant reductions in adult survival and fecundity [16]. When sucrose concentrations in the artificial diet exceed 20%, aphid fluid intake gradually decreases, and levels of 20–40% are detrimental to the growth and development of *M. persicae* [49].

The optimal sucrose concentration for *A*. *pisum* artificial diets ranges from 20–35% [17]. At a 35% sucrose concentration, early-instar nymphs of *A. pisum* exhibit accelerated growth, whereas 20% sucrose enhances survival and fecundity in fourth-instar nymphs and adults [17]. Another study reported consistent results: diets containing 0–5% sucrose are completely unsuitable for *A. pisum* development; diets with 10–20% sucrose support adequate growth, with body weight increasing as sucrose concentration rises; and *A. pisum* exhibits higher fecundity at a 35% sucrose concentration [50]. Furthermore, sucrose concentrations vary widely among different artificial diet formulations, and the optimal concentration must be empirically determined in combination with amino acid ratios and concentration gradients [38].

The optimal sucrose concentration also varies among other aphid species. For *A*. *gossypii*, 20–30% sucrose supports enhanced growth, survival, and reproductive performance. Both nymphs and adults of *A. gossypii* typically exhibit extended survival on diets containing 30–35% sucrose, whereas late-instar nymphs show slightly higher survival rate on diets with 20% sucrose [51]. *S*. *avenae* can survive and reproduce normally over a sucrose concentration range of 10–40%, with survival duration not differing significantly from that of control individuals reared on wheat seedlings. The optimal sucrose concentration for reproductive output is 25%, indicating that *S. avenae* has a broad adaptive capacity to varying sucrose levels [35]. Further research has established that the optimal sucrose proportion in artificial diets for *S. avenae* ranges from 25–30% [52]. *Toxoptera aurantii* (Boyer de Fonscolombe) requires a notably higher sucrose concentration (55%) than most other aphid species [22].

#### 3.1.2. Other Sugars

Besides sucrose, other sugars perform distinct functional roles in aphid physiology and nutrition. In 1970, research demonstrated that melezitose, raffinose, glucose, fructose, galactose, maltose, and trehalose stimulate ingestion and can be metabolized by aphids, based on survival and larviposition rates. Sorbose stimulates ingestion but is not metabolized, whereas mannose, sorbitol, xylose, and ribose neither stimulate feeding nor are utilizable. Rhamnose, arabinose, lactose, and cellobiose directly inhibit fluid uptake [53]. Early-instar *M*. *persicae* were reared on artificial diets in which sucrose was replaced or supplemented with various sugars. In the absence of sucrose, only melibiose and raffinose supported larval growth. With 1% or 2% sucrose, diets containing glucose, fructose, galactose, maltose, trehalose, melezitose, and sorbitol were optimally ingested and efficiently utilized by aphids. Under these conditions, sorbose was poorly utilized, while cellobiose—and presumably lactose—was not utilized at all [53]. Glucose and fructose are critical for carbon nutrition and metabolic regulation in aphids. In *A. pisum*, the fructose moiety of ingested sucrose is highly efficiently assimilated and preferentially respired, whereas the glucose moiety is incorporated into oligosaccharides via the transglucosidase activity of gut sucrase at high sucrose concentrations [48], a process directly linked to osmotic regulation in aphids. Trehalose, a major non-reducing disaccharide in insect hemolymph [54], promotes carbohydrate absorption [55,56] and enhances aphid growth and development [57,58]. Studies have shown that the growth rate of *A. pisum* reared on a diet containing 30% sucrose and 5% trehalose is comparable to that of aphids reared on a diet with 35% sucrose alone [17]. Additionally, high trehalose concentrations in artificial diets increase glucose content in *A. pisum* [54].

#### 3.1.3. Interaction Between Different Carbohydrates

Synergistic interactions can occur among carbohydrates, which means that supplementing diets with multiple carbohydrate sources often improves aphid growth compared with using a single carbohydrate (e.g., sucrose) alone. For example, an artificial diet for *A*. *gossypii* containing a mixture of 20% sucrose and 10% maltose outperformed diets with 20% or 30% sucrose alone and was more conducive to offspring growth [18]. In the artificial rearing of *S*. *avenae*, a 50:50 sucrose–maltose mixture supported optimal rearing performance [59]. Additionally, amino acid concentrations can alter the optimal sucrose concentration in artificial aphid diets [38]. These findings encourage us to pay attention to the interaction between different carbohydrates.

### 3.2. Amino Acids

Amino acids are essential biomolecules that compose the insect body and serve as the material foundation for aphid growth and development. Phloem sap, the natural food source of aphids, typically has a total amino acid concentration of less than 1% [60]. Herbaceous plant phloem sap contains higher nitrogen content than that of woody plants, resulting in faster aphid growth on herbaceous hosts compared to woody hosts [61].

In the phloem sap consumed by *A*. *pisum*, non-essential amino acids account for 50% or more of the total amino acid composition, forming the dominant fraction, whereas essential amino acids constitute a low proportion, rarely exceeding 25% [62]. Natural variations in the amino acid composition of aphid dietary sources alter the reproductive rate of *A. pisum* [62]. In contrast, the essential amino acid content in artificial aphid diets is substantially higher than that in both plant phloem sap and the *A. pisum* body. For specific research purposes, the essential amino acid concentration in such diets can be increased to as high as 55%; elevating essential amino acid concentrations not only elicits consistent feeding behavior in aphids but also compensates for low feeding rates [28,42]. The results showed that during the peak of density of *S. avenae* (Fabricius, 1775), the population level of the amino acids was elevated within infested ears of winter triticale [63]. In shoots of the aphid-infested plants, the amino acid content was reduced, while in the case of root tissues, only the level of nonessential amino acids was decreased and the level of the essential ones was not statistically different from that of the control group [63]. There is a review that focuses on research advances in the metabolomics of plant phloem sap, highlighting its key characteristics: difficulty in sampling and complex composition [64].

#### 3.2.1. Amino Acid Functions

Different amino acids exert distinct functional roles in aphid feeding behavior, development, and reproduction. Aphids require ten essential amino acids: arginine, tryptophan, threonine, valine, phenylalanine, leucine, isoleucine, methionine, histidine, and lysine. Among these ten essential amino acids, only methionine, isoleucine, and histidine are absolutely indispensable for *M. persicae* [65]. Lysine, threonine, and cysteine exert relatively minor effects; their removal from artificial diets retards *M. persicae* growth [65]. Studies have demonstrated that aspartic acid, isoleucine, leucine, methionine, phenylalanine, and tryptophan significantly enhanced the acceptability of sucrose, with methionine exerting a potent effect even at a trace concentration of 0.02% [66]. Amino acid availability is also linked to wing development in *M. persicae*: omission of methionine, isoleucine, or histidine results in a marked increase in the proportion of alate (winged) *M. persicae* nymphs, alongside a significant reduction in nymphal body weight [67].

Methionine and cysteine exert critical roles in the development of *M*. *persicae*. Even at a total amino acid concentration as low as 2%, high methionine concentrations support the normal development of *M. persicae* [65]. Methionine deficiency reduced food intake in *M. persicae* by approximately 50% [68]. A progressive increase in food intake is associated with rising methionine concentrations from 0 to 40 mg/100 mL; no further changes in food intake are observed with progressive increases in methionine concentration up to 320 mg/100 mL [68]. In the absence of cysteine, *M. persicae* growth is impaired, yet compensatory growth can occur via supplementation with inorganic sulfate or additional methionine, indicating that cysteine functions primarily as a sulfur source [65]. Cysteine or histidine deficiency reduces *M. persicae* food intake by 15–33% [68].

Certain amino acids exert synergistic effects on aphid growth and development, which means that some amino acids act as a whole on aphids, and when they are all added or removed from artificial diets, the growth and development of aphids is significantly affected. At conventional concentrations of serine and alanine, aspartic acid, glutamic acid, and their respective amides collectively act as an essential group for sustaining aphid growth [65]. Omitting aspartic acid, glutamic acid, asparagine, and glutamine as a collective group largely inhibits growth, despite the fact that omitting each individual amino acid from this set has no such effect. This group requirement can be partially mitigated by any of the four aforementioned compounds—either alone or in various combinations—or by equivalent concentrations of alanine or serine, but not by glycine [65]. Conversely, supplementing artificial diets with high concentrations of glutamate, alanine, or, most effectively, serine supports growth levels comparable to those observed on diets containing a complete mixture of twenty amino acids [65]. Notably, alate *M. persicae* reared on diets rich in alanine or serine frequently exhibit reddish pigmentation, a phenotype likely associated with the accumulation of metabolic waste products from amino acid catabolism [65]. Studies also showed that the amino nitrogen balance, including the asparagine/glutamine ratio, plays a leading role in wing production in *M. persicae* [69].

Researchers added ^14^C-labeled amino acids in an artificial diet and constructed a metabolism cage for aphids to collect and analyse the radioactivity incorporated into the aphid body. This method was applied to the study of the metabolism of eight energetic amino acids (aspartate, glutamate, glutamine, glycine, serine, alanine, proline, and threonine) in *A. pisum*. All these amino acids except threonine were subject to substantial catabolism, as measured by high ^14^CO_2_ production. The highest turnover was displayed by aspartate, with 60% of its carbons expired as CO_2_. They directly demonstrated the synthesis of three essential amino acids (threonine, isoleucine, and lysine) from the carbons of common amino acids. The synthesis of these three compounds was only observed from amino acids that were previously converted into glutamate [70].

#### 3.2.2. Amino Acid Concentrations in Artificial Diets

Amino acid concentrations exert concentration-dependent effects on aphid physiology and feeding behavior. In artificial diets with 35% sucrose, studies have revealed distinct feeding stimulatory effects of individual amino acids. Leucine and phenylalanine stimulated feeding at concentrations of 0.1–0.75% and up to 1.0%, respectively. Tryptophan and valine acted as phagostimulants at 0.1%, 0.2%, and 0.5%. Threonine showed phagostimulatory activity only at 0.1%. Methionine exerted phagostimulatory effects at 0.05–0.5%. In contrast, histidine and isoleucine displayed no phagostimulatory activity at any concentration tested [71]. Arginine and lysine hydrochloride exert feeding-inhibitory effects on aphids [71]. Among non-essential amino acids, canavanine sulfate and glutamine inhibit *A*. *pisum* feeding at all tested concentrations, whereas homoserine promotes feeding in *A. pisum* at concentrations of 0.1–0.75% [71]. Conversely, arginine, canavanine sulfate, glutamine, histidine, homoserine, isoleucine, leucine, and valine enhance body weight and extend survival duration in *A. pisum*, with leucine playing a key role in nymphal protein synthesis [71]. Lysine hydrochloride, phenylalanine, threonine, and tryptophan neither enhance body weight nor alter survival rates in *A. pisum* [72].

For *M. persicae*, dietary amino acid concentrations below 2% result in a markedly reduced growth rate [16]. Total amino acid concentrations below 1% lead to an extremely low feeding rate in *M. persicae* or cessation of feeding entirely. As dietary amino acid concentrations rise to 3%, the feeding rate increases progressively; an approximate 3% amino acid concentration represents the optimal level for *M. persicae* growth, with further increases in concentration leading to a subsequent decline in feeding rate [73]. *M. persicae* adapted to dietary restriction of amino acids through the upregulation of genes involved in amino acid biosynthesis, glycolysis, and protein degradation, as well as by altering the expression level of genes involved in hormone signaling pathways [74].

For *A. pisum*, the suitable dietary amino acid concentration range is 2–4%, with 3.5% representing the optimal concentration [75]. Feeding rates are minimal in amino acid-free artificial diets. Adult *A. pisum* exhibit the highest feeding rates on diets containing 2.5% amino acids, whereas first- to third-instar nymphs and fourth-instar nymphs achieve peak feeding rates on diets with a 3.5% amino acid concentration [75]. Using deuterium-labeled glutamine, researchers determined that arginine, histidine, isoleucine, leucine, lysine, phenylalanine, threonine, and valine are all biosynthesized by the symbionts of *A. pisum*; these amino acids are therefore classified as non-essential for *A. pisum* in artificial diets [39]. Experimental evidence demonstrates that artificial diets formulated to match the amino acid composition of plant phloem sap are more suitable for supporting the growth, development, and reproductive performance of *A. pisum* [72].

For *A. gossypii*, nitrogen application to cotton plants enhanced both cotton seedling growth and *A. gossypii* performance [76]. The changes in phloem sap were not detected.

### 3.3. Lipids

Research into lipid requirements in aphid artificial diets has centered primarily on steroids. As precursors for hormonal biosynthesis, steroids exert critical roles in aphid developmental physiology [77].

Insects, unlike vertebrates, are unable to synthesize the sterol nucleus [78]. Theoretically, dietary steroid supplementation could enhance aphid development; however, some researchers have proposed that aphid gut symbionts are capable of de novo steroid biosynthesis, obviating the need for exogenous dietary steroid addition. In comparison to *A*. *pisum* reared on natural plant hosts, those reared on artificial diets exhibit a significant increase in neutral lipid levels and a marked decrease in phospholipid content. *A. pisum* accumulate lipids under artificial diet rearing but fail to allocate these lipids toward reproductive processes—a finding that may signal suboptimal nutritional quality of artificial diets. Phospholipids are fundamental structural components of biological membranes. Reduced phospholipid levels in diet-reared aphids may suggest impaired biosynthesis of key cellular components, which indicates that standard artificial diets are poorly suited for the continuous rearing of *A. pisum* [79].

Some researchers have hypothesized that *M*. *persicae* is capable of de novo sterol biosynthesis or that its symbiotic microorganisms supply sterols to the aphid [13]. However, this hypothesis of symbiont-mediated sterol provision was not supported by early investigations. In these studies, artificial diets were supplemented with aureomycin to eliminate symbionts in *M. persicae*, followed by the addition of either lipid extracts from *M. persicae* or cholesterol. Nonetheless, these treatments failed to improve the growth and development of the aphids [80]. Since insect ecdysone biosynthesis relies on sterol precursors [77], it has been hypothesized that sterol deficiency may cause delayed and sometimes incomplete larval development in *M. persicae* [81]. The developmental-promoting effects of sterols were subsequently confirmed experimentally: supplementing artificial diets with cholesterol (2.5 pg/mL) reduced the developmental time of *M. persicae* nymphs to the fourth instar by 12–16% compared with sterol-free diets, with no significant effects on total body weight, fecundity, or offspring birth weight [82]. It has also been demonstrated that symbionts can synthesize sterols required for *M. persicae* embryonic development from dietary precursors. When symbionts were eliminated using chlortetracycline, viable offspring were produced on cholesterol-supplemented diets, whereas embryonic development was arrested before germ band formation on cholesterol-free diets [82].

Contradictory findings, however, have been reported: while sterols are essential for insect development overall, they are not deemed required in artificial diets for *A. glycines*, and cholesterol-containing diets have even been shown to inhibit aphid development [32]. *A. pisum* can also acquire the steroids it requires from its endogenous symbionts [83], and this aphid species can be continuously reared for more than seven generations on cholesterol-free artificial diets [23].

Additionally, *A. pisum* is capable of completing growth and development on sterol-free artificial diets, though dietary sterol supplementation confers additional benefits for aphid growth and development [24]. Supplementing artificial diets with cholesterol benzoate has been shown to increase the survival rate and average fecundity of *A. pisum* [72]. The phloem sap of fava bean (*Vicia faba*) plants utilized by *A*. *pisum* contains three sterols, cholesterol, stigmasterol and sitosterol, in a 2:2:1 ratio. The number and biomass of embryos in aphids on diets with stigmasterol and no sterols were reduced relative to diets with cholesterol or sitosterol, indicating that the reproductive output of *A*. *pisum* can be limited by the amount and composition of dietary sterol [84].

In summary, the necessity of dietary sterol supplementation in aphid artificial diets requires further detailed investigation. For *M*. *persicae* and *A*. *pisum*, most studies indicate that supplementary lipids are not required in artificial diets and that symbionts within these aphids may contribute to lipid biosynthesis. However, given the high diversity of aphid species, whether dietary lipid supplementation is necessary should be evaluated on a species-specific basis. Antibiotic treatment to eliminate symbionts can exert substantial effects on aphids. Investigations into the role of dietary lipid supplementation using this approach are likely subject to multiple confounding factors, which may lead to contradictory conclusions.

### 3.4. Vitamins

Vitamins play critical roles in insect growth and development, as they serve as essential components of intracellular coenzymes and collaborate with enzymes to mediate physiological and biochemical metabolic processes. Water-soluble vitamins are preferred for formulating nutritional solutions for aphids [28]. Studies have demonstrated that *M*. *persicae* requires dietary provision of ascorbic acid (vitamin C) and nine B-complex vitamins: thiamine (vitamin B_1_), riboflavin (vitamin B_2_), nicotinic acid (vitamin B_3_), calcium pantothenate (vitamin B_5_), pyridoxine (vitamin B_6_), biotin (vitamin B_8_), folic acid (vitamin B_9_), meso-inositol (vitamin B-h), and choline [19].

#### 3.4.1. Ascorbic Acid

Ascorbic acid is an essential nutritional component of artificial aphid diets that functions as a feeding stimulant, metal chelator, and antioxidant [72,85]. A growth assay was conducted to determine the relative growth of apterous *M*. *persicae* nymphs initially reared on an ascorbic acid-free diet and subsequently switched to diets supplemented with varying concentrations of ascorbic acid or its analogs. Nymphs achieved maximum body weight at an L-ascorbic acid concentration of 0.2% (200 mg/100 mL diet), whereas dietary ascorbic acid deficiency reduced the aphid reproductive output. L-dehydroascorbic acid, the oxidized form of L-ascorbic acid, exhibited only approximately 50% of the biological activity at an equimolar concentration of L-ascorbic acid, whereas D-araboascorbic acid, a C_5_ epimer of L-ascorbic acid, displayed nearly equivalent biological activity to L-ascorbic acid [86]. Dietary ascorbic acid deficiency significantly reduced the body weight of the parental *A*. *pisum* and induced growth retardation in the F1 generation [72], in addition to decreasing survival rates and causing developmental delays in the F2 generation [85].

In addition to retarding nutrient oxidation, ascorbic acid acts as a chelator, forming stable complexes with mineral elements. Low concentrations of ascorbic acid interact with mineral chlorides, which likely serve as chelators to maintain the ingestion and absorption of trace minerals by aphids [85]. The molecular weights of ascorbic acid and citric acid were substantially lower than that of ethylene diamine tetraacetic acid (EDTA). Complexes formed between mineral elements and these natural ligands are more readily absorbed by aphid intestinal cells than EDTA–mineral complexes, which not only preserves the stability of mineral elements in artificial diets but also enhances their digestion, absorption, and utilization by aphids [87]. Notably, ascorbic acid is highly susceptible to degradation upon dissolution at room temperature; thus, diets supplemented with ascorbic acid are typically stored at −20 °C [19].

#### 3.4.2. B-Complex Vitamins

Generally, insects cannot synthesize the eight B-complex vitamins that serve as coenzymes in a diverse array of essential enzymatic reactions [88]. Most insects acquire B-complex vitamins from dietary sources, symbiotic microorganisms, or a combination [88].

B-complex vitamins exert profound effects on *M*. *persicae*; dietary deficiency of thiamine, nicotinic acid, or calcium pantothenate significantly impairs the growth and maturation of first-generation nymphs, whereas deficiency of any other water-soluble B-complex vitamins delays the growth of first-generation nymphs and prevents successful development of the second generation [19]. The nymphal survival rate of *A. pisum* was not significantly reduced when fed diets deficient in vitamins B_1_, B_3_, B_6_, and B_7_ (which *Buchnera* cannot synthesize), although the corresponding vitamin concentrations in aphid bodies decreased [89].

Thiamine is critical for the survival, development, and reproduction of aphid progeny. Dietary thiamine deficiency did not significantly alter the body weight of *A*. *pisum* but significantly reduced the survival and fecundity of the F1 generation after 10 days [72]. *M. persicae* F1 nymphs were unable to grow and develop under conditions of dietary thiamine deficiency [19]. Thiamine can act on plants to trigger a defense response, thereby conferring aphid resistance [90].

Riboflavin is a non-essential vitamin for aphids that can chelate metal elements in artificial diets, interfere with mineral absorption, and generate toxic metabolites, while imparting a distinct color to the dietary medium. Because of interspecific differences in physiological and biochemical metabolism among aphids, species-specific riboflavin requirements vary substantially. A riboflavin concentration of 0.5 mg/100 mL is optimal for *M. persicae* [19], whereas *A. pisum* requires a markedly higher riboflavin concentration (5 mg/100 mL) to achieve optimal rearing performance—likely due to the sequestration of riboflavin by trace metal elements, including iron, copper, and manganese [25]. Experiments have confirmed that riboflavin concentrations above 0.5 mg/100 mL are detrimental to *M. persicae*, as riboflavin readily forms toxic metal chelates with dietary metal ions [85]. Some researchers have proposed that the primary function of riboflavin in artificial aphid diets is to impart a color cue, whereas the yellow hue of riboflavin combines with green dietary copper ions to produce a yellowish-green color, which mimics plant tissue pigmentation and thereby elicits aphid feeding behavior by capitalizing on chromotaxis [72]. *Arsenophonus*-free *A. gossypii* attained the ability to utilize cucumber after feeding on the riboflavin-free diet, but not on the riboflavin-containing diet, indicating riboflavin and *Arsenophonus*-absent expansion in dietary breadth [91].

Nicotinic acid is an essential vitamin for aphids, and dietary deficiency of nicotinic acid elicits marked reductions in body weight, reproductive rate, and survival rate of the F1 generation of *A. pisum* [72].

Calcium pantothenate modulates the survival and weight gain rates of *A. pisum*, and, in the absence of dietary calcium pantothenate, adult aphids showed a slight reduction in body weight and extremely low survival in the F1 generation. In the F2 generation, aphids barely developed into adulthood, and survival was extremely low after 10 days of feeding [72]. *M. persicae* exhibited a physiological response comparable to that of calcium pantothenate deficiency [19]. The survival rate of aphids decreased significantly when diets lacked B_5_ or contained B_5_ at concentrations ≤ 0.2 mM [89].

Pyridoxine is an essential vitamin for aphids and serves as a biosynthetic precursor of pyridoxal, a compound that mediates the transport and metabolism of tryptophan, cysteine, and serine. Dietary pyridoxine deficiency caused a significant reduction in *A. pisum* body weight and developmental delay at 8 days post-feeding [72].

When artificial diets lack biotin, the F1 generation of *A. pisum* exhibits an elevated reproductive rate relative to the parental generation. However, the resulting nymphs display high mortality within 24 h of hatching, and survival rates are diminished for both the F1 and F2 generations [72].

Folic acid modulates reproductive performance and nymphal weight gain in most insect species [72,92,93]. Dietary folic acid deficiency in *A. pisum* results in reduced weight gain, significantly reduced adult body weight, and severely delayed, impaired adult reproduction (with a 73–78% decline). It also led to an approximately 20% decrease in the survival rate and caused developmental delay and lower body weight in F1 nymphs, accompanied by markedly reduced fecundity [72]. Doubling the dietary folic acid concentration to 2 mg/100 mL resulted in the highest number of live adult *M. persicae* [94]. 4-aminobenzoic acid (PABA) acts as a biosynthetic precursor of folic acid, and deficiency of this compound in artificial diets does not elicit significant alterations in the growth, development, or reproductive performance of *A. pisum* [72].

Myoinositol is a phospholipid that modulates aphid growth and development. Dietary myo-inositol deficiency induced a marked decline in *M. persicae* survival rate, a finding consistent with observations in *A. pisum*, whereas dietary choline deficiency exerted minimal effects on *M. persicae* growth and development [19].

Choline can increase the adult reproduction rate and honeydew excretion [85] and is an essential dietary nutrient for nearly all insect species, which must be acquired from exogenous dietary sources [28,94]. In animal metabolic pathways, choline mediates the biosynthesis of lecithin and acetylcholine and serves as a critical methyl donor [72].

In summary, dietary ascorbic acid (vitamin C) and nine B-complex vitamins at species-specific concentrations play critical roles in the artificial rearing, growth, and development of aphids.

### 3.5. Inorganic Salts

Mineral elements are indispensable nutritional components of artificial diets for aphids. The mineral elements required by aphids include potassium, magnesium, phosphorus, calcium, sodium, iron, manganese, zinc, and copper. The factors that influence mineral bioavailability are the interplay between the absorption sites, minerals, competitors for absorption sites, pH of the local cell surface/lumen region, and presence of mineral sequestering agents [78]. Potassium is involved in numerous chemical reactions and is a component in the structure of many substances, including lipids (phospholipids), some proteins, and nucleic acids [78]. Deficiency of any two of potassium, magnesium, and phosphorus salts in artificial diets results in a complete cessation of nymphal development; deficiency of a single one of these three salts adversely impairs adult survival and nymphal growth [16]. Experimental evidence demonstrates that aphids have elevated requirements for phosphorus, potassium, calcium, and sodium, whereas other trace elements—including iron, manganese, zinc, and copper—must also be present in specific proportions in nutritive solutions [59]. Substituting KH_2_PO_4_ for K_3_PO_4_, calcium lactate for calcium carbonate, and omitting KPO_3_ all yielded favorable rearing performance in aphid culture [59].

#### 3.5.1. Mineral Elements

Aphid species exhibit divergent requirements for dietary mineral element concentrations. For *A*. *pisum*, the optimal concentrations of trace metal cations per 100 mL of artificial diet are as follows: Fe^3+^, 920 µg; Zn^2+^, 400 µg; Cu^2+^, 120 µg; Mn^2+^, 220 µg [24]. In contrast, *M*. *persicae* achieves optimal growth at Fe^3+^ concentrations ranging from 115 to 460 µg per 100 mL, with suboptimal growth observed at an Fe^3+^ concentration of 920 µg per 100 mL [24].

Adequate dietary mineral element concentrations sustain consistent growth and development in aphids. Dietary Fe^3+^ deficiency induces impaired growth and development and reduced fecundity in *A*. *pisum*, with F1 progeny unable to complete ontogenetic development; insufficient Zn^2+^ availability results in a significant reduction in average adult body weight; excessively low Cu^2+^ concentrations not only cause marked declines in average adult body weight and fecundity but also suppress the growth rate of F2 *A. pisum*, leading to mass mortality prior to adulthood. Manganese in artificial diets modulates the average adult body weight and fecundity of *A. pisum* and influences the growth and development of its F2 generation, while the essentiality of Na^+^ for *A. pisum* remains inconclusive [24,72]. For *M*. *persicae*, iron or zinc deficiency results in overt growth impairment in F1 nymphs; manganese deficiency manifests as developmental defects in the F2 generation; and copper deficiency elicits measurable growth effects in the F3 generation. Supplementation of artificial diets with these four trace elements—iron, manganese, copper, and zinc—adequately fulfills the long-term growth and developmental requirements of *M. persicae* [28].

#### 3.5.2. Chelator

To enhance the absorption and utilization of mineral elements—particularly trace elements—by insects, chelating agents are routinely incorporated into artificial diets to chelate trace elements and prevent their precipitation or loss. Commonly employed chelators include EDTA, chloride ions, citric acid, and ascorbic acid. Artificial diets in which cations are chelated with EDTA and analogous compounds fail to support multi-generational growth of *A. pisum* [25]. Investigations with other aphid species, planthoppers, and leafhoppers have also demonstrated that trace elements chelated as chlorides at equimolar concentrations yield rearing performance equivalent to or superior to that of EDTA-based chelation treatments [85]. EDTA exhibits a high affinity for trace elements, and its chelates are resistant to dissociation, thereby increasing the difficulty of mineral element absorption by insects. Additionally, it remains unresolved whether EDTA–mineral complexes, following intestinal absorption, interact with insect midgut epithelial cells or remain localized in the extracellular space as large ligand molecules [28,85].

#### 3.5.3. Sodium Acetate and Sulfur Nutrition

The beneficial effect of sodium acetate supplementation was discovered incidentally during investigations into alarm pheromone production in diet-reared *M. persicae*. *M. persicae* reared on acetate-supplemented artificial diets exhibited increased body size, with individuals attaining dimensions comparable to those of plant-reared conspecifics [95]. Although acetate is known to mediate lipid biosynthesis in insects, the mechanistic basis underlying the beneficial effects of sodium acetate in aphids remains unclear [96].

Since most animals can only assimilate organic sulfur, most research into sulfur nutrition in insects has focused on sulfur-containing amino acids such as methionine and cysteine. Inorganic sulfur routinely incorporated into insect artificial diets is provided as metal cation sulfates (e.g., K^+^ or Mg^2+^ salts). Certain insect species can directly utilize inorganic sulfur via their symbiotic microorganisms [97]. Artificial diets supplemented with cysteine, methionine, and sulfate have been reported to enhance the growth and development of *A. pisum* [49]. Conversely, other studies have proposed that sulfate exerts no discernible effect on the growth and development of *A. pisum* [72,98].

### 3.6. Complex Supplements

Holidic artificial diets often rely on purely chemical formulations, which have certain limitations. The addition of natural nutrients, or their use as substitutes for chemically defined components, can alleviate these drawbacks. This approach provides a more comprehensive nutritional profile for aphids and improves the suitability of such diets for aphid rearing [28]. Common complex nutritional supplements incorporated into aphid artificial diets include yeast/YE, CH, and plant sap extracts.

The yeast has a nutritional composition consisting of approximately 50% protein and nucleic acids, 37–40% carbohydrates, 2–3% lipids, and up to 0.5% water-soluble vitamins [99]. Supplementation of holidic artificial diets with 4–6 g/L natural yeast or nucleotides significantly enhanced the growth and development of *M. persicae*, yielding aphids with uniform developmental and growth trajectories [20]; supplementation with 20 g/L yeast yielded the maximal growth-promoting effect on *A*. *pisum*, with average adult body weight reaching 600–800 µg [49]. A 2.0% YE supplementation can supply *M. persicae* with several essential nutrients that are typically biosynthesized by its symbiotic microorganisms, including adequate choline chloride, biotin, folic acid, myo-inositol, trace minerals, amino acids, and B-complex vitamins, though it is deficient in nicotinic acid, pantothenic acid, pyridoxine, and thiamine. While this supplementation regime sustains multi-generational rearing of *M. persicae*, the resulting aphids exhibit reduced body weight; by contrast, 2.5% enzymatic CH can replace the 20 defined amino acids in holidic diets [30]. Artificial diets containing both YE and CH do not require additional supplementation with trace minerals (copper, iron, manganese, and zinc) [30].

Researchers incorporated boiled, filtered juice pressed from mustard or lettuce into synthetic diets for *M. persicae*, and the optimal rearing effects were observed when mustard juice and lettuce juice constituted 2% and 10% of the total diet volume, respectively; further increases in juice concentration exerted adverse effects on *M. persicae* growth and development [20]. Supplementation of artificial diets with 400–600 mg crude yeast or nucleic acids per 100 mL also enhanced aphid development [20]. Some researchers have proposed that the growth-promoting effects of plant juices stem from their trace metal element content [20].

## 4. Non-Nutritional Factors Affecting the Efficacy of Artificial Diets for Aphids

### 4.1. Suitable pH and Pressure of the Diet

Diverse mechanisms that are pH-related include texture effects and modification of flavors of various nutrient components [78]. Also influenced by pH is the degree of stability of insect diets in relation to microbial contaminants and chemical reactions that are both enzymatic and nonenzymatic [78].

The optimal pH for artificial diets for *A. pisum* is 7.5 [17]. The suitable pH for a diet for *A. gossypii* is 7.4–7.8; as the pH of fresh artificial diets undergoes a slight decline over the course of experimental trials, an initial pH of approximately 7.6–7.8 is near-optimal for this species. *A. gossypii* can acclimate to artificial diets with a pH range of 6.0–8.0 and will even feed and establish populations on diets with a pH of 5.0–9.0 [51]. Notably, *A. gossypii* exhibits a broader tolerance for dietary pH fluctuations and is less sensitive to minor pH variations than *A. pisum*. This interspecific difference is likely attributable to their distinct feeding guilds: *A. gossypii*, a polyphagous species, infests a diverse array of host plant species across distantly related plant families, whereas *A. pisum*—an oligophagous species—has a host range restricted to leguminous plants and displays a marked preference for specific species within this family [51]. *S*. *graminum* exhibits maximal feeding activity at a pH of approximately 8.0 [35,100], while the optimal pH for *S. avenae* artificial diets is 6.0 [35]. A near-optimal pH for the mass rearing of *M*. *persicae* is approximately 7.0 [101].

Plant phloem sap exhibits an inherent hydrostatic pressure. When an aphid’s stylet penetrates the phloem, this sap is forced into the stylet by the endogenous pressure, which facilitates aphid feeding. During feeding, once the stylet pierces a sieve tube, phloem sap flows passively into the stylet, driven by the high hydrostatic pressure of the sieve tube lumen [102]. Accordingly, the application of controlled pressure to liquid artificial diets can facilitate aphid feeding behavior in laboratory rearing systems. Research has demonstrated that applying a pressure of 2 kg/cm^2^ to artificial diets in conjunction with a pressure-resistant composite membrane assembly (three layers of Parafilm plus two layers of gauze) extends the lifespan of *A*. *fabae* [103]. Application of a 0.02 kg/cm^2^ pressure to liquid artificial diets significantly prolongs the lifespan and enhances reproductive output in *M*. *persicae* [104].

### 4.2. Diet Delivery Methods

#### 4.2.1. Aphid Rearing Apparatus

**Figure 2:**
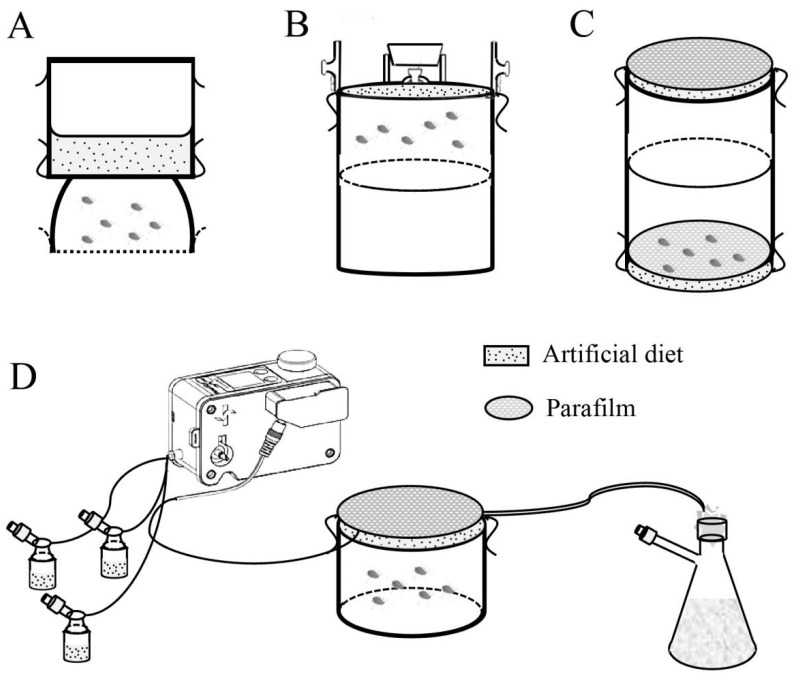
Schematic diagram of aphid rearing devices: (**A**) Artificial rearing device for *M. persicae* (1963) (adapted from [14]), (**B**) multi-generation artificial rearing device for *A. pisum* (1971) (adapted from [25]), (**C**) long-term artificial rearing device for *M. persicae* (2020) (adapted from [29]), (**D**) programmable automated system for continuous rearing of *A. pisum* (1975) (adapted from [105]).

Given aphids’ small body size, piercing-sucking mouthparts, and extraoral digestion, a common aphid rearing approach involves the use of a double-layer parafilm membrane system loaded with an artificial diet. This method was first described by Mittler and Dadd: Twenty *M*. *persicae* individuals were reared in a double-open hemispherical container (32 mm in diameter), with the lower end covered in mesh gauze and the upper end in contact with a diet reservoir. The diet reservoir consisted of a double-open cylindrical container (32 mm in diameter), with both ends sealed with a single layer of parafilm and filled with 2–5 mL of a liquid artificial diet (Figure 2A) [14]. Subsequent aphid rearing protocols have been largely modified and optimized based on this foundational method.

For aphid rearing apparatuses, a multi-layer rearing device employs a glass cylindrical vessel, with the upper section functioning as a diet chamber and the lower section acting as an aphid chamber. The aphid chamber is subdivided into two vertical sections to facilitate the transfer of *A*. *pisum* individuals. Its top is sealed with a single layer of parafilm membrane that interfaces with the diet chamber, and the base of the diet chamber is upwardly curved toward the center, forming a sealed reservoir with the parafilm membrane for diet containment. Diet is introduced from the top via a central aperture in the diet chamber using a syringe, enabling convenient short-term diet replacement without necessitating *A. pisum* to relocate to a new membrane post-replacement (Figure 2B) [25]. Subsequently, automated flow-through rearing systems have been developed for the rearing of *M*. *persicae* and *A. pisum*. Such systems comprise a suite of components: a refrigeration unit, diet reservoirs, a distribution manifold, fluid pumps, and aphid rearing chambers, which enable precise, mechanized regulation of diet delivery. Aphid rearing chambers interface with the diet via parafilm membranes, and fresh diet is circulated through the system at set intervals to displace spent diet, rendering the system suitable for continuous mass rearing of aphids with routine diet replenishment [69,105]. A third rearing method involves the direct connection of two cylindrical vessels: a fresh diet reservoir is linked to a cylindrical rearing vessel previously used for *M. persicae* culture. Spent diet within the rearing vessel is then drained via needle aspiration, facilitating rapid diet replacement; this setup allows for the passive migration of aphids between vessels, minimizing physical trauma to the insects (Figure 2C) [29].

#### 4.2.2. Artificial Diet Membranes

For the selection of artificial diet membranes, researchers have previously tested a variety of membrane materials for aphid rearing, including animal intestinal mesentery (e.g., fish skin or sausage casing) [9], natural rubber [12,106], plant epidermis tissue (e.g., tobacco, tulip, onion) [10], and celluloid [107]; however, none of these materials exhibited rearing efficacy comparable to that of parafilm membranes. Parafilm is supplied as sheets composed of a homogeneous rubber–wax blend and is typically stretched diagonally to its minimum thickness (10–20 μm)—a tenth of its original gauge—for experimental use [10,14,26]. Some protocols involve stretching the membrane nearly to its tensile limit in two perpendicular directions [23]. In consideration of the potential impacts on *A*. *pisum* feeding behavior and growth performance, certain researchers forego stretching parafilm membranes entirely for rearing this species. Preliminary trials indicated no functional difference in rearing performance between stretched and unstretched parafilm membranes [17]. While subsequent researchers validated this finding, stretched membranes are still the standard for aphid rearing due to cost-effectiveness considerations [29].

#### 4.2.3. Diet Sterilization and Automated Feeding System

For artificial diet processing, syringe filtration has been employed for the sterilization of liquid artificial diets [29]. Other researchers have developed automated diet freeze–thaw treatment systems that utilize machine-controlled timed thawing. This setup enables a diet replacement frequency of once every 2–3 days and allows the system to operate continuously for up to 17 days without the need for refilling. Not only do parafilm membranes remain intact for approximately 3 weeks under this regime, but aphids reared in the automated system also feed undisturbed for prolonged periods (2–3 weeks), thus reducing labor and resource inputs (Figure 2D) [105].

In summary, continuous optimization of aphid rearing apparatuses and methodologies has led to the development of a diverse array of techniques tailored to aphid rearing requirements. Future research and development efforts are anticipated to focus on scaling toward large-scale, automated aphid rearing systems.

### 4.3. Feeding Stimulants

Aphids rely on the perception of mechanical and chemical stimuli to select feeding sites during probing and ingestion. No liquid ingestion occurs as aphids pierce the plant epidermis and cortex, nor until their stylets reach the phloem [108]. Nerve receptors at the base or within the stylet likely detect mechanical resistance and variations in osmotic properties, while liquid entering the aphid esophagus constitutes a chemical stimulus, one that is perceived and evaluated by neuronal cells surrounding the pharyngeal region or by the stylet itself [108]. The efficacy of aphid artificial rearing can also be enhanced by recapitulating these mechanical and chemical stimuli in dietary systems.

Common components of aphid artificial diets (e.g., sugars, amino acids, and ascorbic acid) exhibit phagostimulatory activity. Furthermore, species-specific phagostimulants may be required: raffinose for *M*. *euphorbiae* and sorbitol or other taxon-specific stimulants for Rosaceae-feeding aphid species, adaptations linked to the presence of unique phytochemical components in the phloem sap of their respective host plants [109]. Sinigrin acts as a species-specific phagostimulant for *B*. *brassicae* but functions as a feeding repellent for *M. persicae* [104]. The rose aphid (*Marssonina rosae*) will ingest artificial diets containing catechin; this compound acts as a feeding deterrent at concentrations above 0.3 mg/mL yet serves as a phagostimulant at concentrations below this threshold. In vitro assays have demonstrated that the salivary enzymes of this aphid can biochemically convert catechin from a feeding deterrent to a phagostimulant [110].

Aphid feeding behavior is regulated by fluctuations in the concentrations of hemolymph factors, including peptide hormones, biogenic amines, lipids, and carbohydrates, and is primarily mediated by the endocrine system. Initiation or termination of feeding is triggered when the concentrations of these factors reach specific thresholds until their levels return to within the threshold range. This mechanism is consistent with simulated models of feeding cycles [111].

### 4.4. Aphid Digestive Enzymes

**Figure 3:**
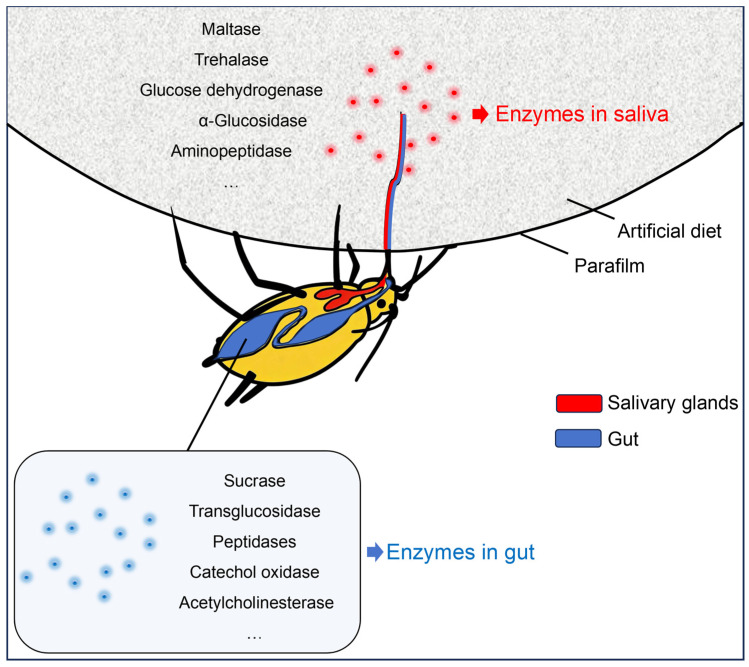
Diagram of intestinal enzymes and salivary enzymes in aphids.

As piercing-sucking hemipterans, aphids depend on salivary enzymes for critical extraoral digestive processes that are indispensable to their feeding physiology. Aphid salivary enzymes are highly diverse and categorized into functional classes, including digestive enzymes, detoxification enzymes, protective enzymes, and hydrolases, which not only mediate plant defense responses but also facilitate aphid feeding and nutrient digestion [112]. Salivary and intestinal enzymes in aphids are diverse. Nutritional components suitable for the digestion of specific aphids can be investigated based on differences in salivary and intestinal enzymes among different aphid species. This study will facilitate the development of artificial diets for various aphid species (Figure 3).

The salivary enzymes present in *M. persicae*, *Megoura viciae*, *A. pisum*, *A. gossypii*, *S. avenae*, and *M. rosae* are shown in Table 2.

Digestive enzymes in saliva—including α-amylase, β-mannosidase, maltase, β-glucuronidase, serine protease, trypsin, cathepsin, and TRE—mediate the degradation of plant cell wall components and plant protein and thus exert a direct digestive role in nutrient breakdown for aphids. Other salivary enzymes, such as ACE, modulate plant physiological processes to facilitate aphid feeding, thereby indirectly supporting aphid nutrient digestion. Collectively, salivary enzymes are pivotal to the digestive physiology of aphids.

Gut enzyme profiles vary significantly among aphid species. The primary proteases in the gut of *A. gossypii* are intracellular cysteine proteases [120] and cytochrome P450 monooxygenases—enzymes that mediate insecticide resistance [121]. *M. persicae* expresses a diverse array of gut enzymes, including acetylcholinesterase, carboxylesterase, glutathione-S-transferase, multifunctional oxidase, superoxide dismutase, catalase, peroxidase, and P450 enzymes [121]. In vitro studies have demonstrated high sucrase activity in the gut of *A. pisum*: the fructose moiety of ingested sucrose is highly efficiently assimilated and likely preferentially allocated to aphid respiratory metabolism. At elevated sucrose concentrations, the transglucosidase activity of gut sucrase catalyzes the synthesis of oligosaccharides from the glucose moiety of sucrose [48]. Additionally, peptidases have been identified in the gut of certain aphid species [122], whereas catechol oxidase and peroxidase have been detected in the gut of *M. rosae* [110].

### 4.5. Aphid Gut Microbiota

Supplementation of artificial aphid diets with a defined concentration of antibiotics enables the selective disruption of symbiotic associations (gut microbiota), thereby facilitating the generation of aposymbiotic aphids. This experimental approach aids in characterizing the role of symbionts in aphid growth and development. For instance, bacteriocytes mediate the conversion of non-essential amino acids in aphid diets into essential amino acids [123] and facilitate the reductive assimilation of sulfate [124]. A previous study presented the first complete genome sequence of *Buchnera aphidicola* strain APS, the obligate endosymbiont of *A. pisum* [125].

Symbionts associated with *M. persicae* facilitate the conversion of ingested ^14^C-acetate to sterols [81]. When *M. persicae* is reared on diets deficient in essential amino acids, its symbionts supply the majority of the essential amino acids required for growth and development, seven of the ten essential amino acid types [32,80]. For instance, methionine is biosynthesized by the bacterial symbionts of *M. persicae* [65].

The high amylase activity detected in the gut extracts and honeydew of *A. pisum* was primarily attributed to microorganisms (bacteria) associated with the aphid gut and honeydew [126]. The biosynthesis of essential amino acids in *A. pisum* is mediated by symbiotic bacteria [39], whereas ammonia, the primary nitrogenous waste product of this aphid, is recycled and utilized by gut bacterial symbionts [127]. Tryptophan synthase activity assays confirmed that *A. pisum* symbionts were capable of de novo tryptophan biosynthesis [128]. Aphid symbionts directly mediate transamination reactions by transferring nitrogen from glutamate or glutamine to a range of amino acids, including several essential amino acids [129,130]. In recent years, genomic and molecular analyses of *A. pisum* symbiotic bacteria have identified numerous genes, nearly 25% of which encode enzymes involved in amino acid biosynthetic pathways, most of which underlie the synthesis of essential amino acids [131]. Symbiotic bacteria also support the homeostatic accumulation of proteins, glycogen, and soluble sugars in *A. pisum* while suppressing aberrant lipid accumulation [132]. All *A. pisum* individuals harbor the vertically transmitted obligate symbiont *Buchnera aphidicola*, which synthesizes and supplies essential amino acids that are deficient in plant phloem sap [133]. Additionally, *A. pisum* may harbor facultative secondary symbionts that are not required for aphid survival or reproduction and confer resistance to a broad range of biotic and abiotic stressors [133].

Genome annotation revealed that *Buchnera* can synthesize only vitamins B_2_ and B_5_ [125]. Transcriptomic analysis revealed that the key gene *panC*, which is involved in vitamin B_5_ biosynthesis in *Buchnera*, exhibited a low abundance of the sense transcript and a high abundance of the antisense transcript. Furthermore, the PanC protein was not detected [89]. It has been speculated that metabolic constraints or antisense transcriptional suppression inhibit vitamin B_5_ biosynthesis.

*Erwinia* inhabits specialized bacteriocytes, is adjacent to *Buchnera*, and co-differentiates with it. *Erwinia* are small and spherical, whereas *Buchnera* are large and spherical. *Erwinia* cannot synthesize essential amino acids and thus depends on *Buchnera*. However, it can produce riboflavin (B_2_) and biotin (B_7_) to compensate for the deficiencies in *Buchnera* and has additionally acquired the ability to synthesize thiamine (B_1_) [134].

The functions of salivary enzymes, intestinal enzymes, and the gut microbiota in aphids are directly associated with aphid feeding and digestion. By investigating the roles of the microbes and enzymes present in aphids, we can selectively increase the content of dietary components suitable for aphid feeding and digestion and reduce that of unsuitable components, thereby modulating the rearing performance of artificial aphid diets.

Similarly, impacts of artificial diets affect not only insects’ gut microbial communities or host metabolomes independently but also microbe–insect interactions [135]. It also represents a significant research direction for the future.

## 5. Summary and Prospects

### 5.1. Environmental Conditions Controlled in Artificial Rearing Systems

In terms of rearing environment, optimal rearing conditions vary among different aphid species.

For *M. persicae*, the optimal temperature is 25 °C [136], the suitable relative humidity ranges from 55% to 90% [137], and the photoperiod is L16:D8 (16 h of light and 8 h of darkness). Moderate temperature fluctuations (e.g., 20 ± 5 °C and 25 ± 5 °C) can significantly improve developmental rate and reproductive success, and *M. persicae* exhibits higher metabolic efficiency and adaptability in variable thermal environments [136].

For *A. pisum* reared on artificial diets in the laboratory, the optimal conditions differed between the color morphs. The green morph has an optimal temperature of 24 °C, optimal relative humidity of 70%, and optimal photoperiod of L:D = 12:12. In contrast, the red morph had an optimal temperature of 20 °C, optimal relative humidity of 70%, and the same optimal photoperiod (L:D = 12:12) [72]. Within a temperature range favorable for the growth and reproduction of *A. pisum*, alternating temperatures resulted in a higher intrinsic growth rate than constant temperatures [138].

For other aphid species, such as *A. nerii*, the optimal rearing temperature is 20 °C. For *A. gossypii*, increasing day length from 6 to 12 to 18 h significantly increased the intrinsic rate of increase, decreased population doubling time, and decreased generation time. However, longevity and net reproductive rate (Ro) were maximized with a 12 h day [139]. The development of *A. gossypii* was the fastest at 30 °C. It achieved its maximum net reproductive number (24.4 nymphs per female) and greatest intrinsic rate of increase (0.386 d^−1^) at 25 °C [140].

### 5.2. Diet Development Strategy

Artificial aphid diets are predominantly formulated as holidic or meridic. These formulations are characterized by complex raw material compositions (more than 20 individual amino acids), cumbersome preparation protocols, and unstable long-term rearing efficacy, often manifesting as population degradation and developmental retardation. Comparison between aphid biological cycle performance on natural host plants versus artificial diets is shown in Table 3. Practical artificial diets, which incorporate complex supplements such as YE or CH, can simplify diet formulations (see the Practical Diet column in Table 1) and have the potential to improve aphid survival rates and fecundity; however, their optimization potential remains largely unexplored.

Future development of practical aphid diets should be integrated with functional studies on the aphid digestive system. By characterizing the activity profiles of salivary enzymes (e.g., maltase, β-mannosidase) and gut enzymes (e.g., cysteine protease, sucrase-transglucosidase), dietary substrates could be precisely matched to the digestive capabilities of target aphid species. For instance, *S*. *avenae* saliva exhibits high maltase activity; thus, supplementation of artificial diets with maltose could enhance energy utilization efficiency. In contrast, the gut of *M. persicae* lacks lactase activity, indicating that lactose-based ingredients should be excluded from the artificial diet.

Furthermore, microbial nutritional compensation mechanisms, such as the synthesis of host-essential amino acids (e.g., methionine and tryptophan) by aphid gut symbionts as well as their participation in sulfur reduction and assimilation, could be strategically leveraged. Appropriate supplementation of artificial diets with inorganic precursors (e.g., sulfate) that are utilizable by symbiotic microbes, coupled with maintaining microbial homeostasis through pH regulation or targeted antibiotic application, is expected to indirectly enhance aphid nutrition and reduce the reliance on exogenous amino acid supplementation.

Advances in omics approaches, analytical chemistry, and precise nutrition could significantly contribute to improving artificial diet formulations and optimizing aphid rearing protocols. The draft genome assembly of *A. pisum* was first presented and it was the first published whole genome sequence of a basal hemimetabolous insect [142]. The genome of the phytotoxic Russian wheat aphid, *Diuraphis noxia* Kurdjumov [143], *R. maidis* [144], and *Sitobion miscanthi* [145] were also sequenced and assembled de novo afterwards. Researchers presented a draft genome sequence of *A. gossypii* and discovered that significantly evolving pathways in the genus *Aphis* are related to biological processes of detoxification, steroid biosynthesis, and ethylbenzene degradation [146]. Feeding experience on artificial diet induced the expansion of the host range of the cucurbit-specialized *A. gossypii*, and this expansion was genotype-specific [147]. A complete genome sequence of the soybean aphid *A. glycines* has been reported. Its extreme host specificity, which restricts feeding on soybeans and Rhamnaceae, is closely associated with these genomic features [148]. Later, the genome of *A. glycines* was reassembled [149]. Aphid genomic analysis provides a key resource for investigating the phenotypic plasticity of aphids [143], plant–aphid interactions [150], host–symbiont cooperation [151], evolutionary patterns of aphid chromosomes [152], and the development of novel pest control strategies [153].

Thanks to the recent advances in mass spectrometry for protein identification, entomologists have greatly benefited from proteomics to unravel the molecular mechanisms behind insect feeding, diapause, metamorphosis, vitellogenesis, and embryogenesis [154]. Current mass spectrometry-based proteomics strategies have enabled researchers to reproducibly, accurately, quantitatively, and comprehensively survey proteome content from cells and tissues to the whole body of an organism [154].

### 5.3. Summary

This study systematically reviewed the core advances in aphid artificial diet research, encompassing rearing methodologies, diet formulations, mechanisms underlying the action of nutritional components, and optimization strategies. Advances in aphid artificial diet development have significantly improved the feasibility of long-term and multigenerational aphid rearing by optimizing rearing techniques and diet formulations.

Among aphid species, *M*. *persicae* and *A*. *pisum* can be reared stably across multiple generations under controlled laboratory conditions and are widely used in pesticide efficacy testing and mass rearing of natural enemies. Additionally, key nutritional factors in artificial diets, including carbohydrates, amino acids, lipids, vitamins, and trace element inorganic salts, exhibit species-specific optimal concentration ranges and collectively regulate aphid growth and development. Furthermore, the optimization of artificial diets can focus on additional parameters such as pH, osmotic pressure, digestive enzyme activity, symbiotic microbial interactions, and environmental conditions.

However, the developmental rate and fecundity of artificially reared aphids remain significantly lower than those of aphids reared on natural host plants, and long-term artificial rearing can induce population degradation. Aphid populations on agricultural crops in temperate regions collapse from peak abundance to local extinction within a few days after midsummer, with ecological drivers, including weather factors, natural enemies, and a decline in plant nutritional quality [155]. Aphids that obligately alternated between host plants annually and those that were agricultural pests exhibited the steepest declines relative to species that were able to persist on the same host plant year-round or in natural areas [156]. Thus, it can be inferred that the aphid population extinction under long-term rearing on artificial diets may result from chronic nutritional deficiencies in the diets, leading to widespread nutritional deprivation and eventual population collapse of the entire aphid colony. Alternatively, nutritional stress may induce aphid migration and increase the production of alate morphs, consequently reducing the number of aphids remaining on the artificial diets over time.

Furthermore, the optimization of aphid artificial diet formulations is mostly based on existing foundational diets, which may create a bottleneck for rearing efficacy. Continued optimization along conventional lines tends to accumulate the inherent drawbacks of original artificial diets, making it impossible to achieve a rearing performance equivalent to that of phloem sap. Therefore, it is essential to explore novel strategies for developing artificial diets. For instance, future diets could be designed by analyzing the composition of phloem sap and aphid hydrolysates to identify the required nutrients and their optimal concentrations. In addition, existing formulations are primarily targeted at model aphid species (*M. persicae* and *A*. *pisum*), whereas diets developed for economically important pest species, such as wheat and cotton aphids, exhibit limited adaptability. This constraint hinders the establishment of standardized artificial rearing methodologies for economically impactful aphid pests.

### 5.4. Prospects

These challenges represent urgent priorities for short-term advancement of research on artificial aphid diets. With the rapid advancement of molecular technologies, such as genetic engineering and genomics, integrating epigenetics and metabolomics is expected to clarify the molecular mechanisms underlying phenotypic degradation, including reduced fecundity and abnormal wing dimorphism, induced by long-term artificial rearing, thereby facilitating targeted diet optimization.

With research advances in aphid salivary enzymes, gut enzymes, gut microbiota, phloem sap composition, and artificial diet nutrients, future studies could leverage the relevant molecular data to precisely design enzyme-responsive diet components and develop novel formulations in a targeted manner. Furthermore, by characterizing the nutritional requirements of different aphid species and leveraging contemporary artificial intelligence (AI) and big data analytics, dynamic regulatory models of the interactions between distinct nutritional components can be developed. No single critical factors seem to be present for generating feeding regulatory mechanisms from several knockout and knockdown experiments, even in insects, indicating that drastic experimental procedures of investigation, such as systemic biological strategies using “comprehensive simulation and computation,” must be undertaken by virtue of perfectly understanding aphid innate feeding behavior in the near future [111]. In addition, image recognition software and artificial intelligence have great potential for the systematic monitoring of insect pests [157]. In the future, it might be worth considering more applications of artificial intelligence in the study of aphids.

In the future, under appropriate conditions, efforts should be directed toward establishing cross-species nutritional requirement databases, developing modular formulation systems, and standardizing rearing environment parameters (e.g., temperature and humidity fluctuation patterns and photoperiod). Additionally, microfluidic diet delivery systems should be developed to reduce rearing costs, simulate the hydraulic pressure of plant phloem, and integrate smart sensing technologies for the real-time regulation of pH and osmotic pressure.

In summary, research on artificial aphid diets has been ongoing for approximately 100 years. Through continuous refinement of diet formulations and innovations in diet development methodologies, artificial aphid rearing technology will become increasingly mature and find broader applications in aphid research and pest management. Breakthroughs in aphid artificial diet research depend on interdisciplinary integration, encompassing in-depth explorations of nutritional physiology and symbiotic interactions for intelligent design, ultimately contributing to green pest control and agricultural ecological security.

## Figures and Tables

**Table 1:** Composition of artificial diets for different aphids (mg/100 mL).

		Diet Developer and Aphid Species											
Nutritional Classification	Chemical Composition (Amount/mg)	Dadd and Mittler, 1966 [13]	Dadd, 1967 [20]	Mittler and Koski, 1976 [30] *		Van Emden and Wild, 2020 [29]	Srivastava and Auclair, 1971 [23]	Akey and Beck, 1971 [25]	Akey and Beck, 1972 [24]	Dadd and Krieger, 1967 [21]	Chawla, Perron, et al., 1974 [31]	Shibao, Kutsukake, et al., 2002 [33,39]	Wille and Hartman, 2008 [32]
		*M. persicae*	*M. persicae*	*M. persicae*		*M. persicae*	*A. pisum*	*A. pisum*	*A. pisum*	*A. fabae*	*M. euphorbiae*	*T. styraci*	*A. glycines*
Carbohydrates	Sucrose	15,000	15,000	15,000 (Meridic)	15,000 (Practical)	15,000	35,000	35,000	35,000	15,000	35,000	29,000	29,000
L-amino acids	Casein hydrolysate (CH)				2500								
	Alanine (Ala)	100	100	100		100	100	100	100	100	100	179	44
	Arginine (Arg)	270	270	270		270	270	400	400	270	270	296	99
	Asparagine (Asn)	550	550	550		550	550	300	300	550	550	298	111
	Aspartic acid (Asp)	140	140	140		140	140	100	100	140	140	88	208
	Cysteine (Cys)	40	40	40		40	40	50	50	40	40	43	27
	Cystine (Cys)							5	5				
	*γ*-amino butyric acid							20	20				
	Glutamic acid (Glu)	140	140	140		140	140	200	200	140	140	149	299
	Glutamine (Gln)	150	150	150		150	150	600	600	150	150	446	535
	Glycine (Gly)	80	80	80		80	80	20	20	80	80	167	16
	Histidine (His)	80	80	80		80	80	200	200	80	80	101	29
	DL homoserine							800	800				
	Isoleucine (Ile)	80	80	80		80	80	200	200	80	80	165	45
	Leucine (Leu)	80	80	80		80	80	200	200	80	80	230	45
	Lysine (mono) hydrochloride	120	120	120		120	120	200	200	120	120	351	95
	Methionine (Met)	40	40	80		40	40	100	100	40	40	72	33
	Phenylalanine (Phe)	40	40	40		40	40	100	100	40	40	170	76
	Proline (Pro)	80	80	80		80	80	100	100	80	80	129	56
	Serine (Ser)	80	80	80		80	80	100	100	80	80	124	85
	Threonine (Thr)	140	140	140		140	140	200	200	140	140	127	114
	Tryptophan (Trp)	80	80	80		80	80	100	100	80	80	43	108
	Tyrosine (Tyr)	40	40	40		40	40	20	20	40	40	39	11
	β-alanyltyrosine											189	
	Valine (Val)	80	80	80		80	80	200	200	80	80	191	73
Vitamins	YE			2 g	2 g								
	Ascorbic acid (vitC)	100	100	100	100	100	100	100	100	100	100	100	100
	Thiamine (vitB1)	2.5	2.5	2.5	2.5	2.5	2.5	2.5	2.5	2.5	2.5	2.5	2.5
	Riboflavin (vitB2)	0.5	0.5			0.5	0.5	5	5	0.5	0.5	0.5	0.5
	Nicotinic acid (vitB3)	10	10	10	10	10	10	10	10	10	10		
	Calcium pantothenate (vitB5)	5	5	5	5	5	5	5	5	5	5	5	5
	Pyridoxine (vitB6)	2.5	2.5	2.5	2.5	2.5	2.5	2.5	2.5	2.5	2.5	2.5	2.5
	Biotin (vitB8)	0.1	0.1	0.1		0.1	0.1	0.1	0.1	0.1	0.1	0.1	0.1
	Folic acid (vitB9)	0.5	0.5	2		0.5	0.5	1	1	0.5	0.5	1	1
	Meso-inositol (vitB-h)	50	50	50		50	50	50	50	50	50	42	42
	Choline chloride	50	50	50		50	50	50	50	50	50	50	50
	Nicotinamide											10	10
	P-aminobenzoic acid (PABA)							10	10			10	10
Inorganic salts	Iron chelates	1.5	1.5	1.1		1.5	1.5	1.336	0.92	1.5	1.5	4.45	4.45
	Zinc chelates	0.8	0.4	1.73		0.8	0.8	0.417	0.4	0.8	0.8	0.83	0.83
	Manganese chelates	0.8	0.8	0.41		0.8	0.8	0.504	0.22	0.8	0.8	0.65	0.65
	Copper chelates	0.4	0.4	0.176		0.4	0.4	0.254	0.12	0.4	0.4	0.47	0.47
	Sodium chloride							1.271	1			2.54	2.54
	Sodium acetate					320							
	KH_2_PO_4_/K_2_HPO_4_·3H_2_O	500	500	1500	1500	750	500	250	250	500	500	250	250
	MgCl_2_·6H_2_O (magnesium chloride·6H_2_O)	200	200				200	200		200	200		
	MgSO_4_·7H_2_O (magnesium sulphate·7H_2_O)			123	123	123			242			242	242
	Cholesterol benzoate							2.5	2.5			2.5	2.5
	Citric acid			10									
	Calcium citrate							10	10			10	10
	Chlorogenic acid										30		
	Water	Make up to 100 mL											
	KOH	For pH adjustment	For pH adjustment					For pH adjustment	For pH adjustment	For pH adjustment		For pH adjustment	
	K_2_HPO_4_·3H_2_O			For pH adjustment	For pH adjustment								
	pH	7	7	6.8	6.8		7.6	7.5	7.5	7	7.6	7.5	7.5

Note: * Except for this column, which corresponds to meridic and practical artificial diets, all other formulations are holidic artificial diets.

**Table 2:** Presence of salivary enzymes in different aphids.

Aphid Species	Enzyme/Protein Name	Classification	Function Description and Literature Citation
*Myzus persicae*	Oxidoreductase	Oxidoreductase	[113]
	Glucose dehydrogenase (GLD)	Digestive enzyme/Effector	[114]
	Glucose oxidase (GOX)	Effector	[115]
	α-Amylase	Digestive enzyme	[115]
	α-Glucosidase	Digestive enzyme	[115]
	Aminopeptidase	Digestive enzyme	[113]
	Metalloprotease	Effector	[113]
	ATP-binding protein	Other	[113]
*Megoura viciae*	Oxidoreductase	Oxidoreductase	[113]
	Peroxiredoxin	Detoxification/Protective	[113]
	Angiotensin-converting enzyme (ACE)	Effector	Interferes with plant physiological processes, promotes aphid feeding [114]
	M1 zinc-dependent metalloprotease	Effector	[113]
	Glucose–methanol–choline oxidoreductase	Effector/Detoxification	[113]
	Regucalcin	Effector	[113]
	Aminopeptidase	Digestive enzyme	[113]
*Acyrthosiphon pisum*	Oxidoreductase	Oxidoreductase	[113]
	Peroxiredoxin	Detoxification/Protective	[113]
	ACE	Effector	Interferes with plant defense, promotes feeding [116]
	M1 zinc-dependent metalloprotease	Effector	Prevents sieve plate clogging [116]
	Glucose–methanol–choline oxidoreductase	Effector/Detoxification	Promotes salivary sheath protein gelation, oxidative detox [116]
	Regucalcin	Effector	Prevents sieve plate clogging [116]
	Aminopeptidase	Digestive enzyme	[113]
	Metalloprotease	Effector	[113]
	GLD	Digestive enzyme/Effector	[113]
	ATP-binding protein	Other	[113]
*Aphis gossypii*	Dipeptidyl aminopeptidase	Digestive enzyme	[117]
	Aminopeptidase	Digestive enzyme	[117]
	Endopeptidase	Digestive enzyme	[117]
	Serine protease	Digestive enzyme	[117]
	Trehalase (TRE)	Digestive enzyme/Effector	Degrades trehalose [117]
*Sitobion avenae*	β-Mannosidase	Digestive enzyme	Degrades plant cell wall hemicellulose, aids stylet penetration [114,118]
	Maltase	Digestive enzyme	Breaks down plant sugars [114]
	β-Glucuronidase	Digestive enzyme	Breaks down plant sugars [114]
	Serine protease	Digestive enzyme	Degrades plant proteins [114]
	Trypsin	Digestive enzyme	Degrades plant proteins [114]
	Cathepsin	Digestive enzyme	Degrades plant proteins [114]
	TRE	Digestive enzyme/Effector	Degrades trehalose, may interfere with plant stress response [114]
	Cytochrome oxidase	Antioxidant enzyme	Metabolizes plant secondary toxins [114]
	Glutathione S-transferase 1 (GST-1)	Antioxidant enzyme	Specifically expressed in salivary glands, binds toxins for excretion [114]
	Esterase	Antioxidant enzyme	Degrades ester toxins (e.g., jasmonic pathway products) [114]
	Peroxidase	Detoxification/Protective	Scavenges plant reactive oxygen species (e.g., H_2_O_2_), inhibits oxidative stress [114]
	β-glucosidase	Effector	Activates release of plant volatile defense compounds [114]
	Phospholipase	Effector	Induces accumulation of plant jasmonic pathway signaling molecules [114]
	GLD	Effector	Replaces GOX function, inhibits plant defense [114]
	ACE	Effector	Interferes with plant physiological processes, promotes aphid feeding [114]
	Calmodulin (CaM) (regucalcin RCN, calumenin CALU)	Effector	Blocks plant sieve tube clogging defense by chelating Ca^2+^ [114]
	Polyphenol oxidase	Effector/Protective	Defense function [119]
*Marssonina rosae*	Catechol oxidase	Effector/Detoxification	Catalyzes catechin oxidation, converting it from feeding inhibitor to phagostimulant [110]
	Peroxidase	Detoxification/Protective	Catalyzes catechin oxidation, converting it from feeding inhibitor to phagostimulant [110]

**Table 3:** Comparison between aphid biological cycle performance on natural host plants versus artificial diets.

Aphid Species	Natural Host Plant Species	Effects of Aphids’ Artificial Feeding (Artificial Diet vs. Plant)	Reference
*Myzus persicae*	*Raphanus sativus*	Aphid growth: 60–80% vs. 100% Mean adult weight: 0.3 mg-0.4 mg vs. 1 mg	[30]
*Myzus persicae*	*Brassica oleracea* var. *gemmifera* Zenker [141]	Population doubling time: 7 d vs. 3 d Mean total fecundity: 16.5 vs. 47	[29]
*Acyrthosiphon pisum*	*Vicia faba* L. [24]	Mean adult weight: 0.8–0.98 mg vs. 2.34 mg	[23]
*Acyrthosiphon pisum*	*Vicia faba* L.	Mean adult weight: 2.06 mg vs. 2.34 mg Average number of progeny: 2.2 vs. 5.5	[24]
*Aphis fabae* infesting *Tropaeolum* plants	*Tropaeolum*	Mean 1st generation of adult aphids’ weight: 0.5–0.6 mg vs. 1 mg	[21]
*Macrosiphum euphorbiae*	*Solanum tuberosum* L.	Average fecundity of apterae: 43 vs. 70	[31]

## Data Availability

No new data were created or analyzed in this study. Data sharing is not applicable to this article.

## Abbreviations

The following abbreviations are used in this manuscript:

CH	Casein hydrolysate
YE	Yeast extract
PABA	P-Aminobenzoic acid/4-aminobenzoic acid
EDTA	Ethylene diamine tetraacetic scid
GLD	Glucose dehydrogenase
GOX	Glucose oxidase
ACE	Angiotensin-converting enzyme
TRE	Trehalase
GST-1	Glutathione S-transferase 1
CaM	Calmodulin
RCN	Regucalcin
CALU	Calumenin
Ro	Net reproductive rate
